# Advances in the study of oral microbiota in association with T2DM: a systematic review

**DOI:** 10.3389/fcimb.2025.1629304

**Published:** 2025-10-01

**Authors:** Mingming Huang, Xinbi Zhang, Leiming Di, Zheng Yi

**Affiliations:** Capital University of Physical Education and Sports, Beijing, China

**Keywords:** oral microbiota, oral microbiology, diabetes, type 2 diabetes mellitus, T2DM, systematic review

## Abstract

**Objective:**

This systematic review aimed to examine the relationship between the oral microbiota and the onset and progression of type 2 diabetes mellitus (T2DM).

**Methods:**

A systematic review was conducted in accordance with PRISMA guidelines. Three independent reviewers searched relevant literature across multiple databases, including PubMed/Medline, Web of Science, and Scopus, covering publications from April 2000 to April 2025.

**Results:**

A total of 1,438 publications were initially identified, of which 34 studies met the inclusion criteria after screening, namely 23 cross-sectional studies and 11 case-control studies. These studies involved 2,062 patients with T2DM and 1,445 non-diabetic controls. All included studies reported a correlation or potential association between the oral microbiota and T2DM. Fifteen studies analyzed alpha diversity, revealing heterogeneous findings: three reported increased diversity in T2DM patients, two reported decreased diversity, and the remainder showed either no significant differences or inconsistent trends. At the phylum level, Firmicutes was consistently elevated in T2DM patients (14 studies), whereas Proteobacteria was often reduced, and findings on Bacteroidetes varied. At the genus level, Streptococcus, Porphyromonas, and Treponema were most frequently enriched in T2DM populations, with Streptococcus significantly elevated in 22 studies. Notably, Porphyromonas gingivalis was repeatedly identified as a potential contributor to systemic inflammation and insulin resistance, indicating a potential pathogenic role in the metabolic dysregulation of T2DM. Species-level analyses further revealed increased abundance of Streptococcus mutans, P. gingivalis, and T. denticola, supporting the hypothesis that oral dysbiosis is linked to T2DM pathogenesis.

**Conclusion:**

There is a significant association between oral microbiota composition and T2DM. These findings highlight the potential importance of oral health monitoring as part of preventive and therapeutic strategies in the management of T2DM.

## 1 Introduction

Diabetes mellitus (DM) is a chronic metabolic disorder characterized by persistent hyperglycemia resulting from insufficient insulin secretion or impaired insulin action, often accompanied by disturbances in glucose, lipid, and protein metabolism (Dale Abel et al., 2024; Yameny, 2024). According to the latest report by the International Diabetes Federation (IDF), 537 million adults worldwide are living with diabetes, a number projected to rise to 783 million by 2045. Type 2 diabetes mellitus (T2DM) is the most prevalent form, accounting for over 90% of all diabetes cases (Schulze and Hu, 2022).T2DM is closely related to obesity (Chandrasekaran and Weiskirchen, 2024), insulin resistance (IR) (Penno et al., 2021), chronic inflammation (Rohm et al., 2022), and oxidative stress (Andreadi et al., 2022; Caturano et al., 2023), which, if not effectively controlled, can lead to complications such as cardiovascular and cerebrovascular diseases, nephropathy, retinopathy, and neuropathy, posing a serious threat to human health (Ali et al., 2022).

The human microbiota plays a significant role in the development and progression of T2DM, involving not only changes in the composition and function of the gut microbiota (Zhou et al., 2022) but also dysbiosis of the oral microbiome (Kumari and Gnanasundaram, 2021). The oral microbiota, as the second most abundant and diverse microbial community in the human body after the gut, comprises approximately 700 microbial species and forms a complex ecological network (Caselli et al., 2020). As a major gateway to the body, the oral microbiota influences both local and systemic health. Its dysbiosis may provoke oral inflammation, compromise mucosal barriers, and allow microbial products into circulation, fueling chronic inflammation and immune imbalance that contribute to diabetes and its complications (Suarez et al., 2020).

In recent years, an increasing number of studies have focused on the association between oral microbiota and diabetes mellitus (Kumari and Gnanasundaram, 2021). For instance, endotoxemia caused by *Porphyromonas gingivalis* infection has been shown to significantly increase the risk of insulin resistance and diabetes in animal models (Li et al., 2024), suggesting that oral microbiota dysbiosis may directly contribute to the development of diabetes. Additionally, several epidemiological studies have demonstrated that the composition of the oral microbiota is closely associated with glycemic control and systemic inflammation in patients with diabetes (Negrini et al., 2021; Zeng et al., 2024). These microbial profiles are further influenced by lifestyle factors, including diet, smoking, oral hygiene practices, and metabolic status (Shaalan et al., 2022; Mohammed et al., 2024).

Given the complex and dynamic nature of the oral microbiota under diabetic conditions, a systematic review is needed to summarize current evidence. This review investigates differences in oral microbiota composition and diversity in individuals with T2DM and explores potential mechanisms by which oral dysbiosis may influence disease onset and progression.

## 2 Materials and methods

### 2.1 Protocol and registration

This systematic review was registered with the International Prospective Register of Systematic Reviews and reported in accordance with the PRISMA statement (Stewart et al., 2015; Chandler et al., 2019)(http://www.crd.york.ac.uk/prospero/, registration number:CRD420251053253).

### 2.2 Eligibility criteria

The literature search strategy was based on the PICOS framework. Population (P): adult individuals diagnosed with T2DM. Intervention (I): assessment of oral microbiota composition in individuals with T2DM. Comparison (C): adult individuals without T2DM. Outcome (O): the association between oral microbiota composition and the presence of T2DM. Study design (S): observational studies examining the association between oral microbiota and T2DM prevalence, including case-control, cohort, and cross-sectional studies. Exclusion criteria: reviews, conference abstracts, case reports, and other publication types that did not provide original data suitable for analyzing the relationship between oral microbiota and T2DM.

### 2.3 Information sources and search strategy

Three independent researchers searched PubMed/MEDLINE, Web of Science, Scopus, and the Cochrane Library using the following keywords: “oral microorganism,” “oral microbiota,” “oral microbiome,” “diabetes,” “saliva microbiota,” “type 2 diabetes,” “cross-sectional study,” and “cohort study.” The search was performed up to April 10, 2025. To ensure research quality, all studies were independently screened and extracted by two reviewers, with disagreements resolved through discussion or adjudication by a third reviewer. The search strategy is shown in **Table 1**.

**Table 1:** Search strategy in each electronic database.

Pubmed
#1: (“Oral Microorganism”[Title/Abstract] OR “Oral Microbiota”[Title/Abstract] OR “oral microbiome”[Title/Abstract] OR “saliva microbiota”[Title/Abstract]) #2: (“Diabetes Mellitus”[MeSH Terms] OR “diabetes”[Title/Abstract] OR “type 2 diabetes”[Title/Abstract]) #3: (“Cross-Sectional Studies”[MeSH Terms] OR “Cohort Studies”[MeSH Terms] OR “Case-Control Studies”[MeSH Terms] OR “cross-sectional study”[Title/Abstract] OR “cohort study”[Title/Abstract] OR “case-control”[Title/Abstract] OR “case control”[Title/Abstract]) #4: #1 AND #2 AND #3

Web of Science
#1: TS=(“Oral Microorganism” OR “Oral microbiota” OR “oral microbiome” OR “saliva microbiota”) #2: TS=(“diabetes” OR “type 2 diabetes”) #3: TS=(“cross-sectional study” OR “cohort study” OR “case-control study” OR “case control study”) #4: #1 AND #2 AND #3

Scope
#1: TITLE-ABS-KEY(“Oral microorganism” OR “Oral microbiota” OR “oral microbiome” OR “saliva microbiota”) #2: TITLE-ABS-KEY(“diabetes” OR “type 2 diabetes”) #3: TITLE-ABS-KEY(“cross-sectional study” OR “cohort study” OR “case-control study” OR “case control study”) #4: #1 AND #2 AND #3

The Cochrane Library
#1: Oral microorganism #2: Oral microbiota #3: oral microbiome #4: saliva microbiota #5: diabetes #6: type 2 diabetes #7: cross-sectional study #8: cohort study #9: case-control study #10: {#1 OR #2 OR #3 OR #4} #11: {#5 OR #6} #12: {#7 OR #8 OR #9} #13: {#10 AND #11 AND #12}

### 2.4 Data collection process

The included studies were analyzed, and data were extracted by two independent researchers. After removing duplicates from the literature search, the selected studies were imported into NoteExpress software. The first round of screening was performed by reviewing titles and abstracts, followed by a full-text review to complete the second round. Extracted data included the first author’s name, country, journal, publication year, sample size, patient age, oral diagnosis, microbiome analysis type, sample extraction, detection methods, and key findings. In cases of disagreement, a third researcher was involved in the decision-making process.

### 2.5 Quality assessment of included studies

The quality of the included studies was assessed using the Joanna Briggs Institute (JBI) critical appraisal checklist, a validated tool for evaluating methodological quality across various study types, including observational and cross-sectional designs. This checklist examines key aspects such as study design relevance, sample selection and size, representativeness, and clarity of data collection procedures.

We also assessed the use of validated measurement tools, potential biases and confounding factors, and the strategies used to control them. The appropriateness and transparency of statistical analyses were evaluated, along with whether ethical approval and informed consent procedures were clearly reported. Based on the number of criteria met, each study was rated as low, moderate, or high quality. These ratings informed the overall strength of the evidence and guided the interpretation of the findings in this review.

## 3 Results

### 3.1 Literature search

**Figure 1:**
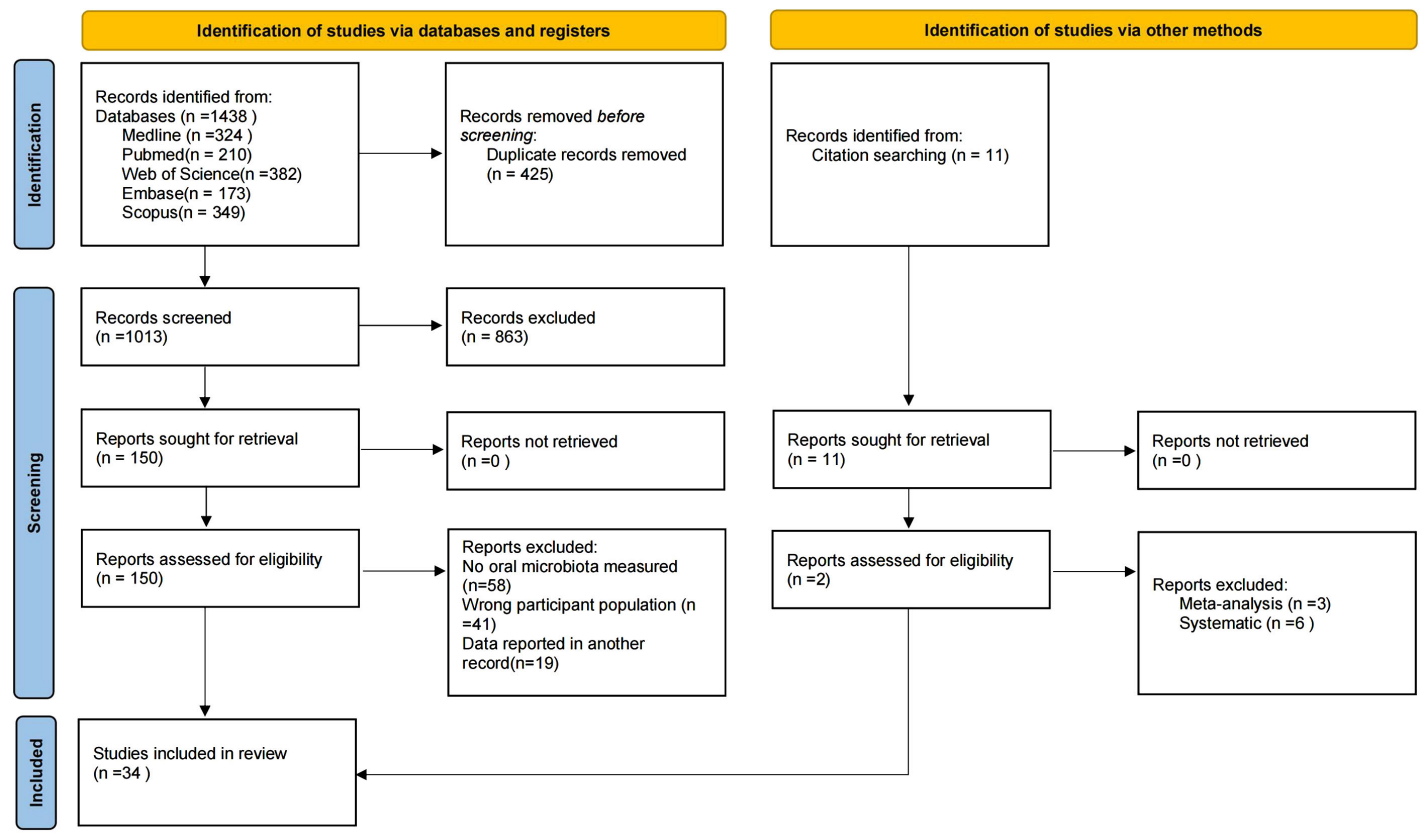
PRISMA flowchart diagram. From Page et al. (2021).

The search strategy is shown in **Figure 1**. After the initial search, a total of 1,438 studies were retrieved from MEDLINE (n = 324), PubMed (n = 210), Web of Science (n = 382), Embase (n = 173), and Scopus (n = 349), with an additional 11 studies retrieved through other methods. After removing duplicates, 1,013 studies remained. Screening of titles and abstracts yielded 150 studies that met the inclusion and exclusion criteria. Following full-text review, 34 studies were finally included (Hintao et al., 2007; Kamaraj et al., 2011; Adeyemi et al., 2019; Kumar et al., 2012; Al Mubarak et al., 2013; Zhou et al., 2013; Cortelli et al., 2014; Kampoo et al., 2014; Kumar et al., 2014; Shenoy et al., 2014; Mohammadi et al., 2016; Rezazadeh et al., 2016; Janem et al., 2017; Long et al., 2017; Ogawa et al., 2017; Schmalz et al., 2017; Hsaine et al., 2018; Latti et al., 2018; Chen et al., 2020; Kori et al., 2020; Matsha et al., 2020; Shi et al., 2020; Sun et al., 2020; Almeida-Santos et al., 2021; Gao et al., 2022; Lu et al., 2022; Sabancı et al., 2022; Guo et al., 2023; Li et al., 2023; Rasouli et al., 2023; Wang et al., 2023; Gu et al., 2024; Soundaram et al., 2024; Tang et al., 2025).

### 3.2 Description of the studies

An overview of study characteristics is provided in **Table 2**. Thirty-four papers published between 2007 and 2025 were included, comprising 23 cross-sectional studies and 11 case-control studies. These studies were conducted in the following countries or regions: China (n=9), India (n=7), United States (n=4), Iran (n=2), Sweden (n=1), Japan (n=1), Thailand (n=1), Germany (n=1), Saudi Arabia (n=1), South Africa (n=1), Portugal (n=1), Turkey (n=1), Pakistan (n=1), Brazil (n=1), and Morocco (n=1).

**Table 2:** Outcomes of the selected studies investigating the association between oral microbiota and T2DM.

Number	Reference	Sample	Measurement method	Microbiota associate with T2DM	Main finding(s)	Alpha diversity analysis
1	Shillitoe et al., 2012	Saliva	16S rRNA	Phyla: Firmicutes↑, Bacteroidetes↔ Genera: Bifidobacteria↓, Fusobacterium↔, Bacteroides thetaiotaomicron↔, Porphyromonas gingivalis↔, Methanobrevibacter ↔ Species:-	- Subjects with T2DM had ~10-fold higher “all bacteria” concentration in oral rinses than the subjects without diabetes -The total amount of “whole bacteria” in the mouth of T2DM group was higher than that of non-diabetic group; - In T2DM patients, Bifidobacteria was significantly reduced in both oral cavity and feces and was correlated with HbA1c > 6.5%	Not reported
2	Latti et al., 2018	Saliva	Culture	Phyla: Firmicutes↔、Proteobacteria↔ Genera: Bifidobacteria↓, Fusobacterium↔, Bacteroides thetaiotaomicron↔, Porphyromonas gingivalis↔, Methanobrevibacter ↔ Species: *Streptococcus mutans*↑	-The amount of Streptococcus mutans in saliva of T2DM group was significantly increased (P< 0.01) -Hyperglycemia was significantly associated with the increase of Streptococcus mutans and other bacteria	Not reported
3	Li et al., 2023	Saliva、supragingival plaque	Metagenomic sequencing	Phyla: Firmicutes↑、Bacteroidetes↑ Genera: Porphyromonas gingivalis ↑、Actinomyces massiliensis ↓、Treponema denticola ↑、Streptococcus mutans↔、Lactobacillus↔ Species: Porphyromonas gingivalis↑、Treponema denticola↑、Aggregatibacter segnis↑、Lactobacillu↔	-T2DM patients without oral diseases exhibited significant dysbiosis in both microbial composition and metabolic profiles; -Periodontal pathogens (P. gingivalis, T. denticola) were significantly elevated and positively correlated with metabolites like cadaverine and n,n-dimethylarginine;	Shannon index:No significant difference Beta diversity :significant difference in supragingival plaque
4	Soundaram et al., 2024	Saliva	Culture	Phyla:- Genera: *Streptococcus*↑, *Lactobacillus* ↑ Species: *Streptococcus mutans*↑, *Lactobacillus*↑	-Diabetic group had significantly higher CFUs of Streptococcus mutans and Lactobacillus; -Diabetic patients showed lower salivary pHand higher DMFS scores;	Not reported
5	Hintao et al., 2007	Saliva, oral rinse, supragingival plaque, subgingival plaque	Culture	Phyla:- Genera: *Streptococcus*↑、*Lactobacillus*↑ Species: Streptococcus mutans↑、Lactobacillus↑、Treponema denticola↑、Prevotella nigrescens↑ Streptococcus sanguinis↑、Streptococcus intermedius↑、Streptococcus oralis↑	-T2DM patients had significantly more root caries and severe periodontitis; -No significant difference in S. mutans, Lactobacillus, or yeasts in saliva between groups; -Supragingival plaque had significantly more cariogenic/pathogenic species in T2DM group; -Root caries was associated with salivary S. mutans, Lactobacillus, and yeasts;	Not reported
6	Ogawa et al., 2017	Saliva	16S rRNA (V4)	Phyla: Bacteroidetes↓ Genera: Actinomyces ↑、Treponema↑、Prevotella↑、Selenomonas ↑、Alloprevotella ↓ Species: Fusobacterium nucleatum↔、Prevotellaintermedia↔	-Bacteroidetes phylum significantly decreased in T2DM; • Dysbiosis observed even in small T2DM sample (n=3); • Highlights potential of salivary microbiota as indicator of systemic health	Shannon index:significantly higher than healthy controls; Beta Diversity:differs significantly
7	Chen et al., 2020	Saliva	16S rRNA (V1–V2)	Phyla: Proteobacteria↓、Firmicutes↑、 Firmicutes/Bacteroidetes (↑ F/B ratio) Genera: Actinomyces ↑、Treponema↑、 Prevotella↑、Selenomonas ↑、Alloprevotella ↓ Species: Fusobacterium nucleatum↔、Prevotella intermedia↔	-T2DM group had significantly different oral microbiota structure vs. healthy controls; -F/B ratio significantly increased in T2DM (7.60 vs. 2.74); -LEfSe analysis identified Neisseria, Haemophilus, Pseudomonas, and Streptococcus enriched in T2DM;	Chao1 index ↑ in T2DM Shannon index ↑ in T2DM T2DM group had significantly higher alpha diversity Beta diversity also significantly different
8	Kampoo et al., 2014	Saliva, supragingival plaque	16S rRNA (V2–V3)	Phyla: - Genera: *Streptococcus mutans*↑, *Lactobacillus*↑ fermentum↑, Actinomyces viscosus↑, Capnocytophaga gingivalis↑, Prevotella multisaccharivorax↑ Species:-	-T2DM patients had significantly higher counts of Streptococcus and Lactobacillus in saliva and plaque compared to controls; -Within T2DM patients, those with active caries had significantly more Lactobacillus in degraded dentine than in plaque; -Bacteroides vulgatus (70%) dominated carious plaque; -S. mutans, L. fermentum, A. viscosus were present in all diabetic patiens;	alpha diversity:No significant difference Shannon index:No significant difference
9	Rezazadeh et al., 2016	Saliva	Culture	Phyla: Streptococcus mutans↑ Genera:- Species:-	-No significant difference in mean levels of S. mutans or Lactobacillus among diabetic dialysis, non-diabetic dialysis, and healthy controls -Positive correlation between S. mutans and FBS in diabetic dialysis group-Positive correlation between S. mutans and BUN (post-dialysis) in non-diabetic dialysis group	Not reported
10	Janem et al., 2017	Saliva	16S rRNA (V3–V4)	Phyla:- Genera: Fretibacterium ↑、Haemophilus↓, Alloprevotella↓, Pseudomonas↓, Lautropia↓ Species:-	-Children with T2D had higher gingival index (p = 0.010) and worse oral health rating; -T2D group had lower dental visit rate in prior 6 months; -Several microbial genera significantly varied even after adjusting for gingival inflammation	Alpha diversity (Shannon, Simpson, Chao): no significant differences; Simpson showed marginal significance Beta diversity:significant differences
11	Kumar et al., 2014	saliva	Culture	Phyla:- Genera: *Candida spp*. ↑ Species:-	-Significant correlation between salivary glucose and oral Candida count in both controlled (r = 0.539) and uncontrolled diabetics (r = 0.743); -High diagnostic sensitivity (83.33%) and specificity (100%) for salivary glucose in detecting T2DM (AUC=0.888, P < 0.001)	Not reported
12	Kumar et al., 2012	Subgingival plaque	Culture	Phyla:- Genera: Peptostreptococcus↑, P. gingivalis↑, Prevotella intermedia↑, A.actinomycetemcomitans↑, Streptococcus sanguis↓ Species:-	-No statistically significant differences in bacterial prevalence between T2DM and control groups (p > 0.05); -P. gingivalis was significantly more prevalent in NIDDM than IDDM (p < 0.05); -High prevalence of periodontopathogens across all groups suggests host factors, not microbiota, may drive disease severity in diabetics	Not reported
13	Schmalz et al., 2017	Subgingival plaque、saliva	PCR	Phyla:- Genera: Porphyromonas gingivalis↑、Parvimonas micra↑、Fusobacterium↑、Campylobacter↑ Species:-	-pH of unstimulated saliva was significantly higher in non-DM group (7.0 vs. 6.7, P < 0.01) -Microbial differences did not translate into worse oral health in DM patients	Not reported
14	Shenoy et al., 2014	saliva	Culture	Phyla:- Genera: *Candida* ↑ Species:-	-Positive candidal growth: T1DM=30%, T2DM=33.3%, Control = 6.7% (P < 0.05) -Significant positive correlation between CFU/mL and FBS (r = 0.571) and HbA1c% (r = 0.596) -CFU/mL positively associated with candidiasis symptoms	Not reported
15	Kamaraj et al., 2011	Subgingival plaque	PCR	Phyla:- Genera: Porphyromonas gingivalis↑、Fusobacterium nucleatum↑、Treponema forsythia↑ Species:-	-No significant differences in PI, GI, GBI, VSC (Tanita), or organoleptic scores between groups -Diabetic group had significantly higher microbial load of Pg and Tf in tongue samples -Fn in tongue significantly correlated with VSC scores (both Tanita and organoleptic) in diabetics	Not reported
16	Adeyemi et al., 2019	Subgingival plaque	culture	Phyla:- Genera: *Actinobacillus*↑、*Streptococcus*↑ Species: Actinobacillus actinomycetemcomitans↑、Staphylococcus aureus↑、Bacteroides oralis↑	-Dental caries and gingivitis more common in non-diabetics; -Diabetics had more Gram-negative anaerobic bacteria (47.6%) and comparable Gram-positive (52.4%) levels; -Suggests higher microbial load and complexity in diabetic-associated oral infections	Not reported
17	Al Mubarak et al., 2013	Subgingival plaque	culture	Phyla:- Genera: *Candida*↑ Species: Candida albicans↑、Candida dubliniensis↑、Candida tropicalis↑、Candida glabrata↑	Overall Candida prevalence in T2DM patients with periodontitis: 52% -Higher prevalence of C. albicans compared to other species -Males and individuals >40 years had more Candida infections	Not reported
18	Mohammadi et al., 2016	Oral swabs and saliva	Culture and PCR	Phyla:- Genera: *Candida*↑ Species: Candida albicans↑、Candida krusei↑、Candida glabrata↑、Candida tropicalis↑	-Diabetic patients had significantly higher oral Candida colonization (P < 0.05) -Candida load (≥50 colonies) significantly more common in T2DM (65% vs. 35.4%) -C. dubliniensis and C. parapsilosis not detected in either group	Not reported
19	Matsha et al., 2020	Subgingival plaque	16S rDNA	Phyla: Fusobacteria↑, Actinobacteria ↑、Proteobacteria ↓ Genera: Actinomyces↑, Corynebacterium↑,Leptotrichia↑, Olsenella↑, Selenomonas↑, Tannerella↑,Prevotella↑、Haemophilus↓, Neisseria↓,Fusobacterium↓, Campylobacter↓, Aggregatibacter↓ Species:-	-Oral microbiome composition varied by glycemic status and periodontal disease status; -Actinobacteria and Fusobacteria significantly increased the odds of DM in patients with gingival bleeding; -Porphyromonas ↑ in DM with gingival bleeding; -In DM + PD ≥4mm, Selenomonas, Leptotrichia ↑; -Streptococcus, Veillonella, Neisseria ↓ associated with reduced DM risk in specific subgroups	Alpha diversity: significant differences Beta diversity :significant differences
20	Guo et al., 2023	Saliva	16S rRNA(V3-V4)	Phyla:- Genus: Prevotella↓, Neisseria↓, Fusobacterium↓,Streptococcus↑,Actinomycetes↑、Porphyromonas↑ Species:-	Oral dysbiosis observed in T2DM. Key oral bacteria such as Streptococcus, Rothia, and Actinomyces were enriched. Actinomyces identified as a key taxon altering microbiota structure. (at genus level)	alpha diversity:Significantly different
21	Wang et al., 2023	Saliva and subgingival plaque	16S rRNA (V2–V4)	Phyla:- Genus: Streptococcus↑、Porphyromonas↑、Prevotellan↑、Fretibacterium↑ Species:-	T2DM patients had distinct oral microbiota with higher periodontal pathogens. Nonsurgical periodontal treatment improved glycemic control and decreased pathogenic taxa.	The alpha and beta diversity: Significantly different bundance :Significantly different
22	Almeida-Santos et al., 2021	Saliva	16S rRNA (V3–V4)	Phyla:- Genus: *Streptococcus*↑、*Prevotella*↑、*TG5*↓ Species: Prevotella intermedia↔、Campylobacterrectus↔、Porphyromonas endodontalis↔、Treponema socranskii↔	No significant difference in overall microbiome between medicated T2DM and controls. TG5 genus more abundant in controls.	Shannon index:No significant differences abundance:No significant differences
23	Sabancı et al., 2022	subgingival plaque	PCR	Phyla:- Genus: Actinobacillus↓、Campylobacter↓、Porphyromonas↓、Tannerella↓、Fusobacterium↓ Species: Fusobacterium nucleatum↓、Campylobacter rectus↓、Porphyromonas gingivalis↓、Tannerella forsythia↓、Treponema denticola↓、Prevotella intermedia↓、Prevotella nigrescens↓	The numbers of T. forsythia, P. gingivalis, and C. rectus species were statistically significantly higher in the control group than the T2DM group in deep pockets	Not reported
24	Zhou et al., 2013	Subgingival plaque	16S rDNA (V1–V3)	Phyla: Actinobacteria↔、Proteobacteria↔、Bacteroidetes↔ Genus: Prevotella↓、Tannerella↓、Pseudomonas↑、Actinomyces↑、Aggregatibacter↑ Species: Treponema denticola↑、Tannerella forsythia↑、Porphyromonas gingivalis↑	In the subjects with healthy periodontium, the abundances of three genera (Prevotella, Pseudomonas, and Tannerella) and nine OTUs were significantly different between diabetic patients and their non-diabetic counterparts.	Not reported
25	Tang et al., 2025	Saliva	16S rRNA (V3–V4)	Phyla:- Genus: *Streptococcus*↑ Species: Capnocytophaga ochracea↑、Treponemasocranskii↑	Oral microbiota varied more by ethnicity and age than by T2DM; distinct keystone genera identified.	Diversity:No significant differences
26	Gu et al., 2024	Saliva	16S rDNA (V3–V4)	Phyla: Firmicutes↓ Genus: *Prevotella*↓ Species:-	Significant microbiota differences across periodontitis stages in T2DM; positive correlations with PD, AL, etc.	Diversity: significantly lower Shannon: not significant.
27	Kori et al., 2020	saliva	16S rRNA(V3–V4)	Phyla: Proteobacteria↓、Firmicutes↑、Bacteroidetes↔、Actinobacteria↔、Fusobacteria↔ Genus: Prevotella↑、Veillonella↑、Leptotrichia↑、Porphyromonas↓、Pseudomonas↓、Stenotrophomonas↓ 、Campylobacter↓ Species:-	Diabetics had reduced diversity and shift toward acidogenic microbiota; gender-specific patterns noted.	Simpson’s and Shannon : significantly lower
28	Cortelli et al., 2014	Subgingival plaque	PCR	Phyla:- Genus: Fusobacterium nucleatum↑、Porphyromonas gingivalis↑、Tannerella forsythia↑、Aggregatibacter actinomycetemcomitans↑、Prevotella intermedia↑、 Campylobacter rectus↑ Species: Fusobacterium nucleatum↑、Porphyromonas gingivalis↑、Tannerella forsythia↑、Aggregatibacter actinomycetemcomitans↑、Campylobacter rectus↑	T2DM patients had higher glucose and lower salivary flow; no difference in pathogen frequency.	Not reported
29	Hsaine et al., 2018	Oral swab	Culture	Phyla:- Genus: Streptococcus↑、Enterococcus↑、Staphylococcus↑、Klebsiella↑、Lactobacillus↑ Species: Streptococcus constellatus↑, Streptococcus acidominimus↑, Streptococcus oralis↑, Enterococcus faecalis↑,Staphylococcus aureus↑, Enterobacter cloacae Klebsiella↑, oxytoca Escherichia coli↑, Pseudomonas aeruginosa↑ and Candida albicans↑	Greater microbial diversity in diabetics; virulent species more prevalent in poorly controlled T2DM.	Not reported
30	Long et al., 2017	saliva	16S rRNA (V4)	Phyla: Actinobacteria↓,、Firmicutes↔ Genus: Actinomyces↓, Atopobium↓,Corynebacterium↓, Mobiluncus↓, Bifidobacterium↓,Rothia↓; Gemella↑ Species:-	High abundance of Actinobacteria, especially Actinomyces and Atopobium, was associated with a significantly decreased risk of T2DM. The abundance of Gemella in Firmicutes phylum was associated with an increased risk of T2DM. The results were generally consistent across racial strata.	Not reported
31	Sun et al., 2020	saliva	16S rRNA (V3–V4)	Phyla: Proteobacteria↓、Bacteroidetes↑ Genus: *Streptococcus*↓、*Prevotella*↑ Species: Porphyromonas gingivalis↑、Treponema medium↑、Prevotella copri↑、Pseudomonas psychrotolerans↓	-DAP patients have significantly increased salivary flora diversity, including inflammation-related flora (Pg, Tf, Td, P. copri, etc.). - After Metformin treatment, the flora structure of DAP patients was more similar to the healthy group	Alpha diversity: no significant difference
32	Shi et al., 2020	Subgingival plaque	Metagenomic sequencing	Phyla:- Genus: Streptococcus↑、Prevotella↑、Porphyromonas↑、Tannerella↑、Treponema↑ Species: P. gingivalis↑, T. forsythia↑, T. denticola↑, F. nucleatum↔, C. rectus↔, P. intermedia↔, S. sanguinis↑, S. gordonii↑, S. oralis↑, Veillonella parvula↑, Rothia dentocariosa↑	- The changes of microbiota in the T2DM group were less than those in the non-diabetic group, but the inflammatory manifestations were similar; -In terms of the function of subgingival flora, butanoate and ascorbate metabolism were up-regulated in T2DM population, suggesting a potential link between T2DM and periodontal disease.	Not reported
33	Lu et al., 2022	saliva	16S rRNA (V3–V4)	Phyla: Fusobacteriota↓, Cyanobacteria↓, and Spirochaetota↓ Genera: Treponema↑, Campylobacter↑, Prevotella↑,Corynebacterium↑, Leptotrichia↑, Selenomonas、Dialister↑, Capnocytophaga↑, Catonella↑, Filifactor↑;Haemophilus↓,Veillonella↓,Streptococcus↓ Species: Campylobacter concisus↑、Prevotella oralis↑、Porphyromonas gingivalis↑、Prevotella intermedia↑	- Spirochaetota represented by Treponema was significantly increased in the TC (T2DM with periodontitis) group. - Alterations in microecology may be associated with changes in oral environment induced by T2DM (e.g., increased glucose content);	Alpha diversity: Shannon significant difference
34	Gao et al., 2022	saliva	16S rRNA (V3–V4)	Phyla: Actinobacteria↑、Firmicutes/Bacteroidetes↑ Genera: Rothia↑、Prevotella_7↑、Veillonella↑,Lactobacillus↑;Streptococcus↓, Neisseria↓ Species:-	- There was no significant difference in the composition of salivary microbiota between T2DM and healthy individuals, but significant differences were observed in the abundance of some key bacteria (such as Rothia).	Alpha diversity: no significant difference

↑ indicates an increase and ↓ indicates a decrease.

Regarding oral microbial detection methods, most studies (n=17) used 16S rRNA high-throughput sequencing technology (Cortelli et al., 2014; Kampoo et al., 2014; Kumar et al., 2014; Rezazadeh et al., 2016; Latti et al., 2018; Chen et al., 2020; Kori et al., 2020; Shi et al., 2020; Sun et al., 2020; Almeida-Santos et al., 2021; Gao et al., 2022; Sabancı et al., 2022; Guo et al., 2023; Rasouli et al., 2023; Wang et al., 2023; Gu et al., 2024; Tang et al., 2025), including Illumina or other high-throughput sequencing platforms; 10 studies used traditional microbial culture methods for strain identification (Hintao et al., 2007; Kamaraj et al., 2011; Kumar et al., 2012; Al Mubarak et al., 2013; Mohammadi et al., 2016; Janem et al., 2017; Long et al., 2017; Ogawa et al., 2017; Schmalz et al., 2017; Li et al., 2023); 5 studies used PCR-related techniques (Adeyemi et al., 2019; Zhou et al., 2013; Shenoy et al., 2014; Hsaine et al., 2018; Matsha et al., 2020); and 2 studies used macro-genome sequencing techniques (Lu et al., 2022; Soundaram et al., 2024). Across all studies, a total of 3,507 subjects were included, comprising 2,062 patients with T2DM patients and 1,445 non-diabetic controls. Sample types included saliva (n=18) (Hintao et al., 2007; Kamaraj et al., 2011; Kumar et al., 2012; Cortelli et al., 2014; Kampoo et al., 2014; Kumar et al., 2014; Janem et al., 2017; Latti et al., 2018; Chen et al., 2020; Kori et al., 2020; Shi et al., 2020; Sun et al., 2020; Gao et al., 2022; Sabancı et al., 2022; Li et al., 2023; Rasouli et al., 2023; Wang et al., 2023; Gu et al., 2024), subgingival plaque (n=9) (Adeyemi et al., 2019; Al Mubarak et al., 2013; Zhou et al., 2013; Mohammadi et al., 2016; Schmalz et al., 2017; Hsaine et al., 2018; Lu et al., 2022; Guo et al., 2023; Tang et al., 2025), oral swab (n=1) (Long et al., 2017), and six studies using multiple sample types (Shenoy et al., 2014; Rezazadeh et al., 2016; Ogawa et al., 2017; Matsha et al., 2020; Almeida-Santos et al., 2021; Soundaram et al., 2024).

### 3.3 Quality assessment and risk of bias of included studies

A total of 34 studies were included in this review, and their methodological quality was assessed using the appropriate Joanna Briggs Institute (JBI) critical appraisal checklists according to study design. Among the included studies, 23 were cross-sectional and 11 were case control. Study designs were determined based on the temporal relationship between exposure and outcome, the presence or absence of follow-up, and the comparative group structure.

According to the JBI quality assessment, 13 studies were rated as high quality (Cortelli et al., 2014; Kampoo et al., 2014; Kumar et al., 2014; Shenoy et al., 2014; Ogawa et al., 2017; Kori et al., 2020; Shi et al., 2020; Sun et al., 2020; Lu et al., 2022; Guo et al., 2023; Wang et al., 2023; Gu et al., 2024; Tang et al., 2025). These were characterized by clearly defined inclusion criteria, representative samples, valid and consistent exposure and outcome measurements, identification and control of confounding factors, and appropriate statistical analyses. The remaining 21 studies were rated as moderate quality (Hintao et al., 2007; Kamaraj et al., 2011; Adeyemi et al., 2019; Kumar et al., 2012; Al Mubarak et al., 2013; Zhou et al., 2013; Mohammadi et al., 2016; Rezazadeh et al., 2016; Janem et al., 2017; Long et al., 2017; Schmalz et al., 2017; Hsaine et al., 2018; Latti et al., 2018; Chen et al., 2020; Matsha et al., 2020; Almeida-Santos et al., 2021; Gao et al., 2022; Sabancı et al., 2022; Li et al., 2023; Rasouli et al., 2023; Soundaram et al., 2024), typically due to limitations in sample size, partial or absent control of confounders, or insufficient reporting of statistical adjustment methods, although most used valid diagnostic and microbiological procedures.

Importantly, study quality appeared to influence the reported findings. High-quality studies tended to show more consistent associations between oral microbiota composition and T2DM, particularly regarding taxa linked with glycemic status. In contrast, several moderate-quality studies yielded heterogeneous or attenuated results, which may be attributable to weaker confounder control, smaller sample sizes, or incomplete adjustment for medication use and comorbidities. This divergence suggests that methodological rigor strengthens the reliability of the evidence base, while limitations in lower-quality studies may partly explain inconsistencies across the literature.

**Figure 2:**
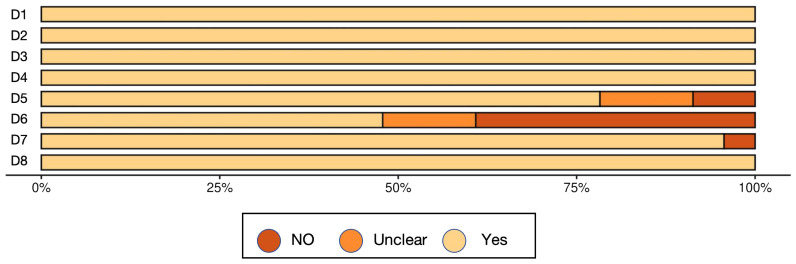
Risk of bias graph 1.

**Figure 3:**
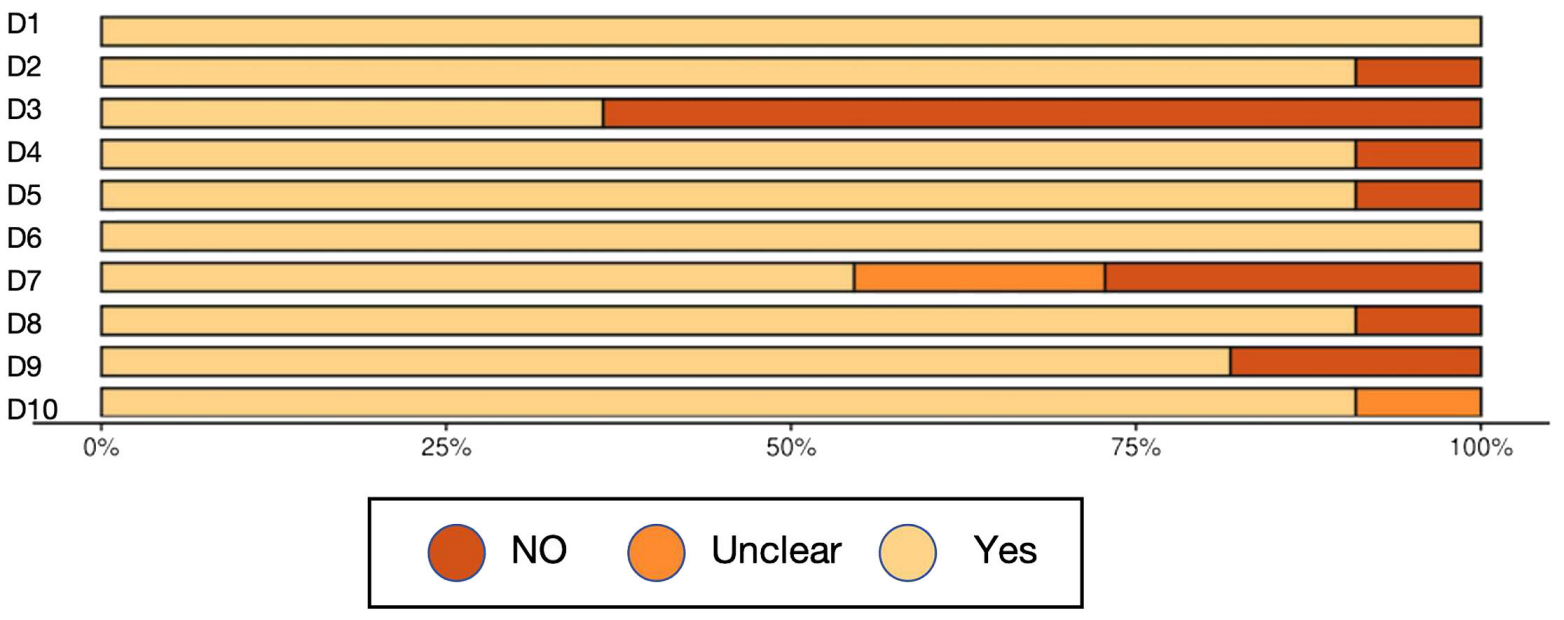
Risk of bias graph 2.

Overall, the methodological quality of the included studies was acceptable, supporting a cautious but meaningful interpretation of the synthesized findings (**Table 3**, **Figure 2**, **Figure 3**, **Table 4**).

**Table 3:** JBI critical appraisal checklist for analytical cross-sectional studies.

Reference	Questions							
	1	2	3	4	5	6	7	8
Latti et al., 2018	Yes	Yes	Yes	Yes	Yes	No	No	Yes
Li et al., 2023	Yes	Yes	Yes	Yes	Unclear	No	Yes	Yes
Soundaram et al., 2024	Yes	Yes	Yes	Yes	No	No	Yes	Yes
Hintao et al., 2007	Yes	Yes	Yes	Yes	Yes	Yes	Yes	Yes
Ogawa et al., 2017	Yes	Yes	Yes	Yes	Yes	No	Yes	Yes
Chen et al., 2020	Yes	Yes	Yes	Yes	Yes	Yes	Yes	Yes
Rezazadeh et al., 2016	Yes	Yes	Yes	Yes	Unclear	Unclear	Yes	Yes
Janem et al., 2017	Yes	Yes	Yes	Yes	Yes	Yes	Yes	Yes
Schmalz et al., 2017	Yes	Yes	Yes	Yes	Yes	Yes	Yes	Yes
Kamaraj et al., 2011	Yes	Yes	Yes	Yes	Yes	No	Yes	Yes
Adeyemi et al., 2019	Yes	Yes	Yes	Yes	No	No	Yes	Yes
Al Mubarak et al., 2013	Yes	Yes	Yes	Yes	Yes	No	Yes	Yes
Matsha et al., 2020	Yes	Yes	Yes	Yes	Yes	Yes	Yes	Yes
Guo et al., 2023	Yes	Yes	Yes	Yes	Yes	Yes	Yes	Yes
Sabancı et al., 2022	Yes	Yes	Yes	Yes	Unclear	No	Yes	Yes
Zhou et al., 2013	Yes	Yes	Yes	Yes	Yes	Yes	Yes	Yes
Tang et al., 2025	Yes	Yes	Yes	Yes	Yes	Yes	Yes	Yes
Gu et al., 2024	Yes	Yes	Yes	Yes	Yes	Yes	Yes	Yes
Kori et al., 2020	Yes	Yes	Yes	Yes	Yes	Yes	Yes	Yes
Hsaine et al., 2018	Yes	Yes	Yes	Yes	Yes	No	Yes	Yes
Sun et al., 2020	Yes	Yes	Yes	Yes	Yes	Yes	Yes	Yes
Lu et al., 2022	Yes	Yes	Yes	Yes	Yes	Unclear	Yes	Yes
Gao et al., 2022	Yes	Yes	Yes	Yes	Yes	Unclear	Yes	Yes

1. Were the criteria for inclusion in the sample clearly defined?; 2.Were the study subjects and the setting described in detail?; 3.Was the exposure measured in a valid and reliable way?; 4.Were objective, standard criteria used for measurement of the condition?; 5.Were confounding factors identified?; 6.Were strategies to deal with confounding factors stated?; 7.Were the outcomes measured in a valid and reliable way?; 8.Was appropriate statistical analysis used?

**Table 4:** JBI critical appraisal checklist for analytical cohort studies.

Author, year	Questions									
	1	2	3	4	5	6	7	8	9	10
Shillitoe et al., 2012	Yes	Yes	Yes	Yes	Yes	Yes	Unclear	NO	Unclear	Unclear
Kampoo et al., 2014	Yes	Yes	NO	Yes	Yes	Yes	Unclear	Yes	Yes	Yes
Kumar et al., 2014	Yes	Yes	NO	Yes	Yes	Yes	NO	Yes	Yes	Yes
Kumar et al., 2012	Yes	Yes	NO	Yes	Yes	Yes	NO	Yes	Yes	NO
Shenoy et al., 2014	Yes	NO	NO	Yes	Yes	Yes	Yes	NO	Yes	Yes
Mohammadi et al., 2016	Yes	Yes	NO	Yes	Yes	Yes	NO	Yes	Yes	Yes
Wang et al., 2023	Yes	Yes	Yes	Yes	NO	Yes	Yes	Yes	Yes	Yes
Almeida-Santos et al., 2021	Yes	Yes	NO	Yes	Yes	Yes	Yes	Yes	Yes	Yes
Cortelli et al., 2014	Yes	Yes	NO	Yes	Yes	Yes	Yes	Yes	Yes	Yes
Long et al., 2017	Yes	Yes	Yes	Yes	Yes	Yes	Yes	Yes	Yes	Yes
Shi et al., 2020	Yes	Yes	Yes	Yes	Yes	Yes	Yes	Yes	Yes	Yes

1.Were the two groups similar and recruited from the same population?; 2.Were the exposures measured similarly to assign participants to both exposed and unexposed groups?; 3.Was the exposure measured in a valid and reliable way?; 4.Were confounding factors identified?; 5.Were strategies to deal with confounding factors stated?; 6.Were the groups/participants free of the outcome at the start of the study (or at the moment of exposure)?; 7.Were the outcomes measured in a valid and reliable way?; 8.Was the follow-up time reported and sufficient to be long enough for outcomes to occur? 9. Was follow up complete and, if not, were the reasons to loss to follow-up described and explored? 10. Were strategies to address incomplete follow-up utilized? 11. Was appropriate statistical analysis used?

### 3.4 Oral microbiota and T2DM

A total of 34 studies were included in this systematic review, spanning 2007 to 2025 and covering findings from multiple countries. These studies revealed common features of oral microecological disorders in patients with T2DM. Most reported that the total bacterial load in the oral cavity of patients with T2DM was significantly higher than that in non-diabetic controls. In particular, flora associated with dental caries and yeast infections, such as Streptococcus mutans, Lactobacillus spp., and Candida albicans, showed significant enrichment in the T2DM population. In addition, salivary acidification and decreased pH often showed a synergistic trend with changes in microbial community structure.

Of the 34 included studies, 15 analyzed the Alpha diversity of oral flora in patients with T2DM (Cortelli et al., 2014; Kampoo et al., 2014; Kumar et al., 2014; Rezazadeh et al., 2016; Chen et al., 2020; Kori et al., 2020; Shi et al., 2020; Almeida-Santos et al., 2021; Gao et al., 2022; Sabancı et al., 2022; Guo et al., 2023; Rasouli et al., 2023; Wang et al., 2023; Gu et al., 2024; Soundaram et al., 2024). Overall, α-diversity findings were heterogeneous. Three studies (Kampoo et al., 2014; Chen et al., 2020; Wang et al., 2023) reported significantly higher Alpha diversity in patients with T2DM, suggesting that their oral flora may exhibit a more complex or disorganized structure. In contrast, two studies (Cortelli et al., 2014; Kori et al., 2020) found a significant decrease in Alpha diversity, manifested by reductions in the Shannon and Simpson indices, suggesting impaired richness or homogeneity of the flora. Three additional studies noted statistically significant differences in α-diversity without a clear directional trend, showing complex variations across subgroups (Kumar et al., 2014; Almeida-Santos et al., 2021; Soundaram et al., 2024). No significant differences were reported in the remaining six studies (Kumar et al., 2014; Rezazadeh et al., 2016; Shi et al., 2020; Sabancı et al., 2022; Rasouli et al., 2023; Gu et al., 2024). Overall, T2DM not only alters the number and composition of oral microorganisms but may also disturb oral microecological homeostasis by affecting the local metabolic environment, pH, and salivary flow rate. These alterations may increase the risk of oral diseases such as caries and periodontal disease (**Table 5**).

**Table 5:** General characteristics of the selected studies investigating the association between oral microbiota and T2DM.

Number	Reference	Country	Study design	Study sample size	Diabetes/metabolic status	Report on association between microbiota/periodontal status	Statistical analysis
1	Shillitoe et al., 2012	USA	Case control	N=13 (T2 DM) N=16 (Healthy Adults)	T2DM	Yes	Yes
2	Bhagyashri Ramachandra Latti, 2016	India	Cross-sectional pilot study	N=30 (T2 DM) N=30 (Healthy Adults)	T2DM	Yes	Yes
3	Li et al., 2023	china	Cross-sectional pilot study	N=10 (T2 DM) N=10 (Healthy Adults)	T2DM	Yes	Yes
4	Soundaram et al., 2024	India	Cross-sectional pilot study	N=30 (T2 DM) N=30 (preT2 DM) N=30 (Healthy Adults)	T2DM	Yes	Yes
5	Hintao et al., 2007	Sweden	Cross-sectional pilot study	N=105 (T2DM) N=103 (Healthy Adults)	T2DM	Yes	Yes
6	Ogawa et al., 2017	Japan	Cross-sectional pilot study	N=3 (T2DM) N=12 (Non-T2DM) N=9 (Healthy Adults)	T2DM	Yes	Yes
7	Chen et al., 2020	China	Cross-sectional pilot study	N=280 (T2DM) N=162 (Healthy Adults)	T2DM	Yes	Yes
8	Kampoo et al., 2014	Thailand	Cross-sectional pilot study	N=10 (Without active caries T2DM) N=10 (active caries T2DM) N=11 (Healthy Adults)	T2DM	Yes	Yes
9	Rezazadeh et al., 2016	Iran	Cross-sectional pilot study	N=30 (diabetic dialysis patients) N=28 (NO-diabetic dialysis patients) N=27 (Healthy Adults)	T2DM-dialysis	Yes	Yes
10	Janem et al., 2017	America	Cross-sectional pilot study	N=16 (T2DM) N=14 (Obese) N=19 (Healthy Adults)	T2DM	Yes	Yes
11	Kumar et al., 2014	India	Case control	N=30 (Controlled T2DM) N=30 (Uncontrolled T2DM) N=30 (Healthy Adults)	T2DM	Yes	Yes
12	Kumar et al., 2012	India	Case control	N=15 (insulin-dependent diabetes mellitus patients) N=15 (noninsulin-dependent diabetes mellitus) N=15 (adult periodontitis patients without diabetes)	T2DM	Yes	Yes
13	Schmalz et al., 2017	Germany	Cross-sectional study	N=66 (T2DM) N=93 (Healthy Adults)	T2DM	Yes	Yes
14	Shenoy et al., 2014	India	Cross-sectional study	N=30 (T2DM) N=30 (Healthy Adults)	T2DM	Yes	Yes
15	Kamaraj et al., 2011	India	Cross-sectional study	N=15 (T2DM-periodontitis) N=15 (periodontitis)	T2DM	Yes	Yes
16	Adeyemi et al., 2019	India	Case control	N=62 (T2DM) N=38 (Healthy Adults)	T2DM	Yes	Yes
17	Sultan Al Mubarak, 2012	Saudi Arabia	Cross-sectional study	N=42 (T2DM-periodontitis)	T2DM	Yes	Yes
18	Mohammadi et al., 2016	Iran	cross-sectional study	N=58 (T2DM) N=48 (Healthy Adults)	T2DM	Yes	Yes
19	Matsha et al., 2020	South Africa	cross-sectional study	N=32 (Pre-T2DM) N=32 (T2DM) N=32 (T2DM receiving treatment) N=30 (Healthy Adults)	T2DM	Yes	Yes
20	Guo et al., 2023	China	Cross-sectional	N=183 (T2DM) N=74 (Healthy Adults)	T2DM	Yes	Yes
21	Wang et al., 2023	China	Case-control	N=11 (T2DM) N=11 (Healthy Adults)	T2DM	Yes	Yes
22	Almeida-Santos et al., 2021	Portugal	Case-control	N=25 (T2DM) N=25 (Healthy Adults)	T2DM	Yes	Yes
23	Sabancı et al., 2022	Turkey	Case-control	N=14 (T2DM) N=12 (Healthy Adults)	T2DM	Yes	Yes
24	Zhou et al., 2013	China	Case-control	N=7 (NO T2DM +NO CP) N=8 (T2DM+ CP) N=8 (NO T2DM+ CP) N=7 (T2DM+ NO CP)	T2DM	Yes	Yes
25	Tang et al., 2025	China	Case-control	N=37 (T2DM) N=25 (Healthy Adults)	T2DM	Yes	Yes
26	Gu et al., 2024	China	Case-control	N=30 with T2DM and Stage I periodontitis N=30 with T2DM and Stage II periodontitis N=30 with T2DM and Stage III periodontitis N=30 with T2DM and Stage IV periodontitis	T2DM	Yes	Yes
27	Kori et al., 2020	Pakistan.	Case-control	N=49 (T2DM) N=55 (Healthy Adults)	T2DM	Yes	Yes
28	Cortelli et al., 2014	Brasil	Case-control	N=49 (T2DM) N=55 (Healthy Adults)	T2DM	Yes	Yes
29	Hsaine et al., 2018	Morocco	Case-control	N=33 (T2DM)poorly controlled N=33 (T2DM)controlled N=68 (Healthy Adults)	T2DM	Yes	Yes
30	Long et al., 2017	USA	Case-control	N=98 (T2DM) N=99 (T2DM-obesity) N=97 (Healthy Adults)	T2DM	Yes	Yes
31	Sun et al., 2020	China	Cross-sectional study	N=9 (T2DM) N=27 (Healthy Adults)	T2DM	Yes	Yes
32	Shi et al., 2020	USA	Case-control	N=15 (T2DM) N=16 (Healthy Adults)	T2DM	Yes	Yes
33	Lu et al., 2022	China	Cross-sectional study	N=10 (T2DM+periodontitis) N=10 (periodontitis) N=16 (Healthy Adults)	T2DM	Yes	Yes
34	Gao et al., 2022	USA	Cross-sectional study	N=273 (T2DM) N=197 (Healthy Adults)	T2DM	Yes	Yes

#### 3.4.1 Phylum level

A total of 14 studies analyzed phylum-level changes in the oral flora of patients with T2DM (Cortelli et al., 2014; Kampoo et al., 2014; Janem et al., 2017; Latti et al., 2018; Chen et al., 2020; Kori et al., 2020; Shi et al., 2020; Sun et al., 2020; Gao et al., 2022; Guo et al., 2023; Li et al., 2023; Rasouli et al., 2023; Soundaram et al., 2024; Tang et al., 2025). Firmicutes was the phylum most frequently associated with T2DM, reported in all 14 studies, and consistently showed an increasing trend. Bacteroidetes, a common oral phylum, was mentioned in eight studies (Cortelli et al., 2014; Kampoo et al., 2014; Latti et al., 2018; Chen et al., 2020; Shi et al., 2020; Rasouli et al., 2023; Soundaram et al., 2024; Tang et al., 2025). Findings varied, with some studies reporting a decrease in abundance, while others found no significant changes. Proteobacteria were mentioned in five studies, with most reporting a decreasing trend (Cortelli et al., 2014; Kampoo et al., 2014; Shi et al., 2020; Guo et al., 2023; Tang et al., 2025). In addition, Fusobacteria and Actinobacteria were each reported in two studies (Cortelli et al., 2014; Guo et al., 2023), wher eas other phyla, such as Cyanobacteria and Spirochaetota, were only rarely mentioned.

#### 3.4.2 Genus level

All 34 included studies analyzed changes in the oral flora of patients with T2DM (Hintao et al., 2007; Kamaraj et al., 2011; Adeyemi et al., 2019; Kumar et al., 2012; Al Mubarak et al., 2013; Zhou et al., 2013; Cortelli et al., 2014; Kampoo et al., 2014; Kumar et al., 2014; Shenoy et al., 2014; Mohammadi et al., 2016; Rezazadeh et al., 2016; Janem et al., 2017; Long et al., 2017; Ogawa et al., 2017; Schmalz et al., 2017; Hsaine et al., 2018; Latti et al., 2018; Chen et al., 2020; Kori et al., 2020; Matsha et al., 2020; Shi et al., 2020; Sun et al., 2020; Almeida-Santos et al., 2021; Gao et al., 2022; Lu et al., 2022; Sabancı et al., 2022; Guo et al., 2023; Li et al., 2023; Rasouli et al., 2023; Wang et al., 2023; Gu et al., 2024; Soundaram et al., 2024; Tang et al., 2025) at the genus level. Overall, significant alterations were observed, closely linked to both the pathophysiological characteristics of T2DM and changes in the oral microenvironment. Streptococcus was the genus most significantly affected, reported in 18 publications, with most showing an increased abundance (Hintao et al., 2007; Al Mubarak et al., 2013; Kampoo et al., 2014; Rezazadeh et al., 2016; Janem et al., 2017; Long et al., 2017; Ogawa et al., 2017; Schmalz et al., 2017; Shi et al., 2020; Almeida-Santos et al., 2021; Gao et al., 2022; Lu et al., 2022; Sabancı et al., 2022; Li et al., 2023; Rasouli et al., 2023; Wang et al., 2023; Gu et al., 2024; Soundaram et al., 2024). Prevotella was mentioned in 16 studies, but results were heterogeneous: some reported an increase (Cortelli et al., 2014; Rezazadeh et al., 2016; Ogawa et al., 2017; Schmalz et al., 2017; Chen et al., 2020; Shi et al., 2020; Almeida-Santos et al., 2021; Gao et al., 2022; Lu et al., 2022; Sabancı et al., 2022; Guo et al., 2023; Rasouli et al., 2023), while others reported a decrease (Kumar et al., 2014; Kori et al., 2020; Tang et al., 2025). Porphyromonas was reported in 10 studies, with most showing increased abundance in patients with T2DM, suggesting a potential role in T2DM-associated oral dysbiosis (Adeyemi et al., 2019; Zhou et al., 2013; Cortelli et al., 2014; Shenoy et al., 2014; Hsaine et al., 2018; Latti et al., 2018; Almeida-Santos et al., 2021; Lu et al., 2022; Wang et al., 2023; Soundaram et al., 2024). Fusobacterium and Treponema were each reported in seven studies (Adeyemi et al., 2019; Zhou et al., 2013; Shenoy et al., 2014; Hsaine et al., 2018; Latti et al., 2018; Guo et al., 2023; Wang et al., 2023), and both were predominantly found in elevated abundance. Overall, the frequent occurrence and altered abundance of these genera suggest that T2DM may drive the oral flora toward increased pathogenicity through changes in the oral environment.

#### 3.4.3 Species level

Of the 17 studies analyzing species-level changes in the oral flora (Hintao et al., 2007; Al Mubarak et al., 2013; Zhou et al., 2013; Mohammadi et al., 2016; Long et al., 2017; Ogawa et al., 2017; Hsaine et al., 2018; Chen et al., 2020; Matsha et al., 2020; Shi et al., 2020; Gao et al., 2022; Lu et al., 2022; Sabancı et al., 2022; Li et al., 2023; Gu et al., 2024; Soundaram et al., 2024; Tang et al., 2025), Porphyromonas gingivalis was the most frequently reported species, appearing in seven studies (Zhou et al., 2013; Hsaine et al., 2018; Latti et al., 2018; Shi et al., 2020; Gao et al., 2022; Soundaram et al., 2024; Tang et al., 2025), all of which showed an increasing trend. Streptococcus mutans was the next most frequently reported species, appearing in six studies (Hintao et al., 2007; Rezazadeh et al., 2016; Janem et al., 2017; Ogawa et al., 2017; Latti et al., 2018; Soundaram et al., 2024), and also generally showing an increasing trend. Treponema denticola was mentioned in four studies, mainly with increasing trend (Zhou et al., 2013; Ogawa et al., 2017; Soundaram et al., 2024; Tang et al., 2025). Fusobacterium nucleatum was reported in three studies (Zhou et al., 2013; Hsaine et al., 2018; Chen et al., 2020), but findings varied. Campylobacter rectus was reported in two studies (Hsaine et al., 2018; Sabancı et al., 2022), with inconsistent results: some showed increased abundance, while others reported a decrease or no significant change.

In summary, different taxonomic levels of oral flora showed specific patterns of change in patients with T2DM. Species such as Firmicutes (phylum), Streptococcus spp. (genus), and *S.* mutans (species) showed a consistent trend of elevation across most studies, suggesting a close relationship with the onset and progression of T2DM. However, some bacterial groups exhibited heterogeneous trends across studies, which may reflect differences in sample characteristics, detection techniques, and study design. Further high-quality studies are needed to confirm these associations.

## 4 Discussion

T2DM is a rapidly growing chronic metabolic disease worldwide, with more than 500 million patients currently affected by metabolic disease and T2DM accounting for over 90% of these cases (Rasouli et al., 2023). In the human oral cavity, the complex and diverse microbial community has a profound impact on health. Oral flora interacts with host metabolic status through multiple mechanisms, including inflammatory responses, insulin resistance, and immunomodulation (Lee et al., 2021).When oral microorganisms enter the systemic circulation via swallowing or gingival microdamage, they may serve as risk factors for triggering or exacerbating metabolic disorders (Jia et al., 2023).Currently, routine screening for T2DM relies mainly on blood glucose testing, which has limitations, especially during the early asymptomatic stage (Ortiz-Martínez et al., 2022). In recent years, oral microecology, due to its close association with glucose metabolism, has been regarded as an auxiliary screening and early risk prediction tool (Liu et al., 2021). The relative abundance of certain salivary taxa has been shown to significantly differentiate T2DM from nondiabetic individuals with good sensitivity and specificity (Shrivastava et al., 2025). Notably, oral flora is more stable in the short term and easier to sample than intestinal flora, making it a promising source of noninvasive biomarkers. Focusing on oral samples, this review aimed to summarize the characteristic changes of oral flora in patients with T2DM and to deepen the understanding of the possible relationship between T2DM and oral health.

A total of 34 studies with 2,062 patients with T2DM were included. Fourteen studies demonstrated correlations between specific phyla of oral microbiota and T2DM (Cortelli et al., 2014; Kampoo et al., 2014; Janem et al., 2017; Latti et al., 2018; Chen et al., 2020; Kori et al., 2020; Shi et al., 2020; Sun et al., 2020; Gao et al., 2022; Guo et al., 2023; Li et al., 2023; Rasouli et al., 2023; Soundaram et al., 2024; Tang et al., 2025). All of the 34 studies examined associations between specific genera and T2DM (Hintao et al., 2007; Kamaraj et al., 2011; Adeyemi et al., 2019; Kumar et al., 2012; Al Mubarak et al., 2013; Zhou et al., 2013; Cortelli et al., 2014; Kampoo et al., 2014; Kumar et al., 2014; Shenoy et al., 2014; Mohammadi et al., 2016; Rezazadeh et al., 2016; Janem et al., 2017; Long et al., 2017; Ogawa et al., 2017; Schmalz et al., 2017; Hsaine et al., 2018; Latti et al., 2018; Chen et al., 2020; Kori et al., 2020; Matsha et al., 2020; Shi et al., 2020; Sun et al., 2020; Almeida-Santos et al., 2021; Gao et al., 2022; Lu et al., 2022; Sabancı et al., 2022; Guo et al., 2023; Li et al., 2023; Rasouli et al., 2023; Wang et al., 2023; Gu et al., 2024; Soundaram et al., 2024; Tang et al., 2025), and 17 analyzed species-level changes in patients with T2DM (Hintao et al., 2007; Al Mubarak et al., 2013; Zhou et al., 2013; Mohammadi et al., 2016; Long et al., 2017; Ogawa et al., 2017; Hsaine et al., 2018; Chen et al., 2020; Matsha et al., 2020; Shi et al., 2020; Gao et al., 2022; Lu et al., 2022; Sabancı et al., 2022; Li et al., 2023; Gu et al., 2024; Soundaram et al., 2024; Tang et al., 2025). Notably, 14 studies reported significant differences in oral microbiota composition between patients with T2DM and healthy controls (Hintao et al., 2007; Al Mubarak et al., 2013; Kampoo et al., 2014; Kumar et al., 2014; Shenoy et al., 2014; Mohammadi et al., 2016; Rezazadeh et al., 2016; Ogawa et al., 2017; Hsaine et al., 2018; Latti et al., 2018; Chen et al., 2020; Almeida-Santos et al., 2021; Guo et al., 2023; Wang et al., 2023), suggesting that certain oral microorganisms may be associated with an elevated risk of developing T2DM. In terms of phylum, several studies reported significant differences in the abundance of Firmicutes, Bacteroidetes, and Proteobacteria compared to healthy controls (Cortelli et al., 2014; Shi et al., 2020; Tang et al., 2025). At the genus level, significant differences were reported for Streptococcus, Prevotella, and Porphyromonas in patients with T2DM compared to healthy individuals. In addition, Streptococcus emerged as an important focal point, with 18 studies demonstrating its association with the progression of T2DM (Hintao et al., 2007; Al Mubarak et al., 2013; Kampoo et al., 2014; Rezazadeh et al., 2016; Janem et al., 2017; Long et al., 2017; Ogawa et al., 2017; Schmalz et al., 2017; Shi et al., 2020; Almeida-Santos et al., 2021; Gao et al., 2022; Lu et al., 2022; Sabancı et al., 2022; Li et al., 2023; Rasouli et al., 2023; Wang et al., 2023; Gu et al., 2024; Soundaram et al., 2024), highlighting its relevance in this area of research. In terms of diversity, 15 studies analyzed the Alpha diversity of oral flora in patients with T2DM (Cortelli et al., 2014; Kampoo et al., 2014; Kumar et al., 2014; Rezazadeh et al., 2016; Chen et al., 2020; Kori et al., 2020; Shi et al., 2020; Almeida-Santos et al., 2021; Gao et al., 2022; Sabancı et al., 2022; Guo et al., 2023; Rasouli et al., 2023; Wang et al., 2023; Gu et al., 2024; Soundaram et al., 2024), with some heterogeneity in the results. Three studies reported significantly higher diversity (Kumar et al., 2014; Almeida-Santos et al., 2021; Soundaram et al., 2024), while no significant differences were observed in six studies (Kumar et al., 2014; Rezazadeh et al., 2016; Shi et al., 2020; Sabancı et al., 2022; Rasouli et al., 2023; Gu et al., 2024). These discrepancies may reflect the influence of multiple underlying factors. Periodontal status may act as an important effect modifier, as active inflammation can alter microbial community richness and evenness in divergent ways depending on disease severity and treatment history (Griffen et al., 2012). Similarly, glycemic control has been shown to shape oral ecological conditions: poorly controlled hyperglycemia favors the dominance of acidogenic taxa and may lead to reduced diversity, whereas in some cases the concurrent colonization of opportunistic species could manifest as apparent diversity gains (Latti et al., 2018). In addition, the use of antidiabetic medications, particularly metformin, has been associated with shifts in microbial composition through both immunomodulatory and metabolic pathways, further contributing to heterogeneity across studies (Gu et al., 2021). Beyond these biological influences, methodological factors such as sampling site, sequencing depth, and the choice of diversity indices may also have contributed to the variability observed.

At the phylum level, 14 studies analyzed changes in patients with T2DM. Firmicutes consistently showed a higher abundance and was the phylum most closely associated with T2DM. In contrast, Proteobacteria showed a significant decrease in several studies, while Bacteroidetes displayed inconsistent trends, with some studies reporting reduced abundance and others finding no significant differences. These results are consistent with previous findings; for example, Tokman et al. reported that an increase in Firmicutes may be strongly associated with chronic low-grade inflammation and metabolic disturbances in patients with T2DM (Bahar-Tokman et al., 2022).Some strains in this phylum can produce metabolites such as short-chain fatty acids during carbohydrate metabolism. These metabolites may not only alter oral ecology by affecting the mucosal barrier and local pH but also modulate host immune responses and insulin sensitivity, thereby promoting diabetes progression (Molinsky et al., 2025).

At the genus level, the oral flora of patients with T2DM showed changes in the abundance of several key taxa. Notably, Streptococcus was repeatedly reported to be elevated in most studies. This genus is widely present in normal oral ecology and has a strong ability to metabolize sugar, rapidly proliferating and producing acidic metabolites in high-sugar environments, thereby lowering oral pH and promoting the development of dental caries and periodontal disease (Ali et al., 2021).Patients with T2DM provide favorable conditions for Streptococcus enrichment due to decreased salivary flow rate and altered salivary composition, which may constitute an important mechanism driving the oral flora toward pathogenicity. In addition, the genus Porphyromonas (particularly P. gingivalis) also showed a trend of increased abundance in several studies (Gu et al., 2024). P. gingivalis is a key causative agent of periodontal disease and represents the core of the “oral–systemic inflammatory axis.” It secretes virulence factors such as lipopolysaccharides (LPS) and proteases that activate the host immune system and induce systemic inflammatory responses, which in turn may promote insulin resistance (Reyes, 2021; Murugaiyan et al., 2024). Its enrichment in the T2DM population suggests that this genus may not only contribute to oral disease but may also participate in the systemic regulation of T2DM progression. Prevotella is another genus frequently reported in T2DM studies, though its trends vary. Some studies found an increase in its abundance in the oral or intestinal flora of patients with T2DM, potentially linked to its ability to ferment carbohydrates and produce butyrate (Zhang et al., 2021). In contrast, other studies reported a decrease. These differences may reflect ecological roles of Prevotella in different oral sites, disease stages, or comorbid contexts and may also be influenced by factors such as diet and oral hygiene.

At the species level, changes in specific bacteria provide more precise insights into the relationship between oral flora and T2DM. Streptococcus mutans, the main causative agent of dental caries, was significantly increased in patients with T2DM. The hyperglycemic environment favors the growth of S. mutans, and the acidic substances it produces further damage tooth enamel, creating a vicious cycle (Brito et al., 2021). Similarly, periodontal pathogens such as Porphyromonas gingivalis and Treponema denticola were also increased in most studies. These species exacerbate systemic inflammatory responses by triggering periodontal inflammation and releasing inflammatory mediators, which in turn impair glycemic control and insulin sensitivity (Liu et al., 2024). Although trends for other species such as Fusobacterium nucleatum were less consistent, their role in oral dysbiosis should not be overlooked. *F. nucleatum* may aggravate oral disease progression by promoting biofilm formation and enhancing the invasiveness of other pathogenic bacteria.

Research indicates that metabolic dysregulation in diabetes mellitus (DM) exacerbates inflammation and promotes microbial dysbiosis in the subgingival microbiome, which is a key factor in the progression of periodontitis in diabetic patients. Hyperglycemia elevates glucose levels in saliva, providing a nutrient-rich environment for cariogenic bacteria in the dental biofilm. Studies have shown that saliva in patients with DM contains higher levels of glucose, urea, and total protein, while exhibiting lower calcium levels and acidic pH (Verhulst et al., 2019), which further support the growth of pathogenic bacteria. These dysbiotic shifts in the oral microbiome are not only associated with local tissue destruction but also contribute to systemic inflammation. Cytokines such as interleukin-1e (IL-1leu), tumor necrosis factor-s (TNF-r-s), and the receptor activator of nuclear factor κa ligand (RANKL) have been implicated in mediating periodontitis in diabetic patients. Additionally, interactions between advanced glycation end products (AGEs) and their receptor (RAGE) exacerbate inflammation and periodontal tissue destruction (Mealey and Oates, 2006; Taylor et al., 2013). Recent studies suggest that diabetes may enhance the pathogenicity of the oral microbiome through IL-17-mediated pro-inflammatory mechanisms. These immune disruptions in DM lead to dysbiosis in the subgingival microbiome, predisposing individuals to periodontitis. Moreover, these dysbiotic shifts may also affect the gut microbiome via the oral–gut pathway, contributing to systemic inflammation and insulin resistance, thereby linking oral health to broader metabolic dysfunction in diabetic patients (Li et al., 2023).

Emerging evidence suggests that antidiabetic medications, particularly metformin, may influence hostuence,lys.r interactions beyond glucose regulation. Metformin has been shown to modulate the gut microbiota by enriching beneficial taxa such as *Akkermansia muciniphila* and other short-chain fatty acid producers, while reducing potentially pathogenic bacteria. Although direct evidence of its impact on the oral microbiota is limited, preliminary findings indicate distinct microbial signatures in patients with T2DM receiving metformin therapy, possibly mediated by reduced systemic inflammation, improved immune balance, and altered salivary metabolic profiles. In contrast, evidence regarding the microbiome-related effects of other antidiabetic drugs, such as DPP-4 and SGLT2 inhibitors, remains scarce. Longitudinal and interventional studies are needed to clarify whether these agents exert protective, neutral, or adverse effects on the oral microbial ecosystem and metabolic outcomes (Hung and Hung, 2020).

This study has several methodological limitations. Different detection methods (e.g., PCR, 16S rRNA gene sequencing) significantly influence the interpretation of results, as each technique has distinct advantages and inherent biases. PCR can precisely amplify target microbial DNA but is restricted to known species and may overrepresent certain taxa, whereas 16S rRNA sequencing provides broader community profiles but with limited resolution and lower sensitivity for low-abundance microbes. These methodological differences may contribute to inconsistencies in reported abundance and limit comparability across studies. In addition, most included studies were cross-sectional in design, which restricts causal inference. Another important limitation is the inconsistent handling of key confounders, including oral hygiene, diet, smoking, and metformin use. Quality assessment revealed that 21 studies did not adequately control for these factors, which may introduce systematic bias and obscure whether observed microbial changes are attributable to T2DM itself or to external influences. To address these issues, future studies should adopt prospective cohort designs, use standardized microbiome sequencing technologies, and apply rigorous statistical methods to control for confounders. Considering lifestyle, metabolic status, and oral environmental factors will further strengthen the validity and reliability of conclusions.

## 5 Conclusions

This systematic review identified significant changes in the oral flora of patients with T2DM across 34 studies. These changes were observed at the phylum, genus, and species levels, with the most consistent increases reported for the phylum Firmicutes, the genus Streptococcus, and the species Porphyromonas gingivalis. Total oral bacterial load was generally higher in patients with T2DM, while bacterial diversity showed heterogeneous patterns across studies. Given the strong association between oral flora and T2DM, future research should prioritize clarifying causal relationships. In addition, maintaining good oral hygiene may contribute to both the prevention and management of diabetes.
